# Geographical heterogeneity in the analysis of factors associated with leprosy in an endemic area of Brazil: are we eliminating the disease?

**DOI:** 10.1186/s12879-015-0924-x

**Published:** 2015-04-25

**Authors:** Mônica Duarte-Cunha, Geraldo Marcelo da Cunha, Reinaldo Souza-Santos

**Affiliations:** Department of Health Surveillance, Municipal Health Secretariat, Duque de Caxias, Rua James Franco, 3, Duque de Caxias, Rio de Janeiro 25215-260 Brazil; Department of Epidemiology, National School of Public Health Oswaldo Cruz Foundation, Rua Leopoldo Bulhões, 1480, Rio de Janeiro, Rio de Janeiro 21041-210 Brazil; Department of Endemic Diseases Samuel Pessoa, National School of Public Health Oswaldo Cruz Foundation, Rua Leopoldo Bulhões, 1480, Rio de Janeiro, Rio de Janeiro 21041-210 Brazil

**Keywords:** Leprosy, Epidemiology, Disease control

## Abstract

**Background:**

The leprosy transmission chain is very complex and, in order to intervene in this transmission, more must be known about the factors linked to falling ill. There are doubts as to the influence of population size, population density and the disease’s magnitude in detection rate trends. This paper aimed to identify factors associated with detection of leprosy in an endemic municipality of Rio de Janeiro State, Brazil.

**Methods:**

This ecological study in Duque de Caxias municipality, Rio de Janeiro State, Brazil, used neighbourhoods (*bairros*) as the unit of analysis. Selecting new cases of leprosy detected from 1998 to 2006, the analysis examined clinical, socioeconomic and service variables using a Poisson log-Normal model.

**Results:**

In the municipality overall, 2572 new cases were detected, a rate of 3.70 cases/10,000 inhabitants. The results describe a heterogeneous distribution of cases and rates in the municipality. The final model displayed a significant association with indeterminate clinical form (β = 2.599), proportion of homes with running water (β = -2.334) and presence of a decentralised health care unit (β = 0.524).

**Conclusion:**

Although the results indicate progress towards elimination of the disease in the municipality, high rates continue to be detected in municipal sub-regions. The following question can thus be posed: over how wide a geographical area could the disease be thoroughly eliminated, given this heterogeneity within a small municipality?

## Background

Although the clinical manifestations of leprosy is well known, it is still endemic in some parts of the world, such as India and Brazil [1]. This is partly due to incomplete knowledge about the disease’s full transmission cycle and the main risk factors involved, which interferes in disease control.

Hosts have the potential to infect much more frequently than they show symptoms, due to the bacillus’s low pathogenicity [2]. Individuals may transmit the bacillus for a long period before the first symptoms begin, that is, in a period of subclinical incubation. Therefore, prevention of transmission cannot rely only on early diagnosis and treatment [3]. Household contacts play an important role in transmission of the disease, and these individuals constitute the group with the highest risk of falling ill [4-6].

Serological tests can now identify possibly bacilliferous individuals [7-9]. These tests have the potential for use in primary health care, not only to classify cases as paucibacillary or multibacillary, but also to identify individuals at higher risk of becoming ill, and to predict higher risk of recurrence [10]. On the other hand, PCR (Polymerase Chain Reaction) methods seem promising for identifying “apparently” healthy individuals [11-13]. Identification of the group of people who can really be considered bacilliferous, regardless of whether or not they are ill, is essential to interrupting the chain of transmission.

In countries such as Ethiopia and Indonesia, where leprosy is endemic and the polychemotherapy (PCT) scheme has been in place for about 15 years, over 5% of the population carry *Mycobacterium leprae* DNA. This suggests that the disease cannot be eliminated as a public health problem simply by PCT treatment [14].

There are doubts about the influence of population size, population density and local endemicity level on world detection rate trends [15]. As for the microenvironment, other factors must be considered, such as the number of people per household and per room of the house, precarious sanitation conditions, genetic susceptibility, low level of schooling, local region social and cultural dynamics and others [16,17].

When examining leprosy’s disappearance from Norway around 1920, long before effective drugs against mycobacterium were used, the disease’s decline was observed to coincide with local economic growth. Also, reduction in detection rates was found to be accompanied by greater detection of multibacillary forms in older individuals [3].

**Figure 1:**
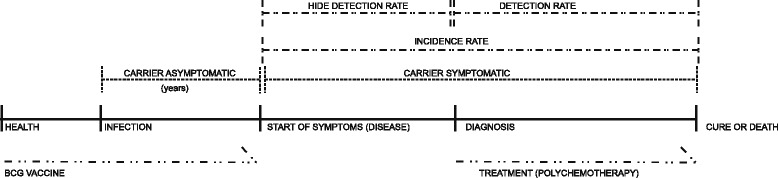
Diagram of the transmission cycle of Leprosy.

Earlier diagnosis could certainly help to interrupt the disease’s transmission cycle and this could lead to substantial reduction in the number of cases [18]. The model describing the leprosy transmission cycle (Figure 1) recognises that it seems to be influenced by unknown factors, creating barriers to the disease’s global elimination [19,20].

The hypothesis that asymptomatic carriers participate in the transmission chain deserves special attention, since this cannot be identified clinically in current routine primary health care.

Difficulty arises in using detection rates in leprosy epidemiology, because the incubation period (whether asymptomatic or oligosymptomatic) is very long, in most cases preventing discovery of the exact moment of symptom onset (Figure 1). Accordingly, limitations must be considered when the detection rate is analysed. The earlier the diagnosis, the closer the detection rate will be to the incidence rate, as the time elapsed between symptom onset and diagnosis is shortened [21]. To ascertain whether the two rates are drawing closer to each other, data can be used to indicate this earliness, such as the increase in the number of cases with indeterminate clinical form or the reduction in cases with grade II physical disability. This could improve the reliability of using detection-rate based analysis.

Brazil, despite having substantially reduced the prevalence rate, still has a high detection rate and a large proportion of cases with physical disability (15%), higher than either China, India or Thailand (3%, 2.5% and 4%, respectively) after 2010 [22]. Rates are highest in Brazil’s North, Northeast and Mid-West regions in 2009 [23].

Essential to control and achieving the elimination target is to identify the various risk factors involved in contracting the disease, because this enables the bacillus’ behaviour and persistence in different regions to be understood. This study aimed to identify factors associated with detection rate of leprosy, taking into account local region socioeconomic, service and clinical-epidemiological differences.

## Methods

This ecological study took as its unit of analysis the neighbourhoods (*bairros*) of Duque de Caxias municipality, Rio de Janeiro State. The municipality, which lies within the Rio de Janeiro metropolitan area in southeast Brazil, has a population estimated in 2010 at 855,042 and an area of 442 km^2^ (http://www.cidades.ibge.gov.br/xtras/perfil.php?lang=&codmun=330170&search=rio-de-janeiro|duque-de-caxias, accessed on 10 Nov 2010).

New cases of leprosy, from 1998 to 2006, in residents in the region were geocoding by neighbourhood identification code, based on the National Notifiable Conditions System (SINAN) database and IBGE. This period was chosen based for database excellent quality and low level of underreporting. Before geocoding process, all neighborhoods identification code were checked with the names of the neighborhoods. If in doubt, the address record was conferred on Google Maps to confirm the neighborhood of residence. Therefore, there was no loss of records.

Socioeconomic Information from 2000 demographic census of the Brazilian Geography and Statistics Institute (IBGE) and the health service data from the Leprosy Programme of Duque de Caxias Municipal Health Office, for the period from 1998 to 2006, were aggregated by neighbourhood. Lastly, the covariates used in the analysis were divided into two subgroups:

1. Covariates relating to clinical-epidemiological factors from SINAN: male-female case ratio; multibacillary-paucibacillary case ratio based on operational classification; ratio of grade II physical disability cases to the sum of grade I and 0 cases; and ratio of cases with indeterminate clinical form to the sum of cases with clinical tuberculoid, dimorphous and lepromatous forms. These variables were created from the clinical and operational classification, used by the Brazilian health service and existing in SINAN. The classifications are based on the criteria of the World Health Organization and the VI International Congress of Leprosy, Madrid, 1953 [24-27];
2. Covariates relating to socioeconomic and health service factors: number of referral health care facilities with a dermatologist and care provided by the Leprosy Programme; number of primary health care facilities with Family Health Programmes and care provided by the Leprosy Programme; number of local case-tracking campaigns; proportion of households where the head is illiterate; proportion of households where the head earns up to 1 minimum wage; proportion of households where the head is unemployed; population density; proportion of households with running water; proportion of households with running water in at least one room; proportion of households connected to sewerage system; proportion of households with no toilet; proportion of households with seven or more residents; and proportion of households that dispose of waste in vacant lot.

The digital map of the municipality was obtained from the website of the IBGE (http://www.ibge.gov.br/home/estatistica/populacao/default_censo_2000.shtm accessed on 08 Jun 2010), as was the population of each neighbourhood, which was derived from the census tracts of the 2000 census through operations between layers in the Geographical Information System.

The data analysis used a Poisson log-Normal model assuming the numbers of cases *y*_*i*_ observed in each neighbourhood as realisations of Poisson random variables *Y*_*i*_ with means μ_*i*_ = *E*_*i*_θ_*i*_ conditional on the underlying relative risks θ_*i*_ and the expected number of cases *E*_*i*_. The log-transformed risk can be written as

$$ \log \left({\theta}_i\right)={\beta}^{\hbox{'}}{x}_i+{u}_i, $$

where *x*_*i*_ are the covariates, β the corresponding vector of coefficients and *u*_*i*_ is a normally-distributed random variable with mean 0 and varianceσ_*u*_^2^, to explain the possible extra variation in the data. This model has the advantage of being easily extended to a conditional autoregressive model (CAR) by including a spatially-structured error *v*_*i*_on the right side of the equation above [28],

$$ \log \left({\theta}_i\right)={\beta}^{\hbox{'}}{x}_i+{u}_i+{v}_i. $$

The spatially structured term *v*_*i*_, is also normally distributed, but its values for different areas are spatially dependent. Specifically, the mean of *v*_*i*_, given the values of term *v*_*j*_, for *j* ≠ *i*, is the average of the *v*_*j*_at the *n*_*i*_ areas deemed to be neighbours of the ith area, whilst its variance is ${\sigma}_v^2/{n}_i$.

A Bayesian approach was taken and non-informative prior densities were assigned to all given parameters: β_*i*_ (normal prior distribution) , σ_*u*_^2^ and σ_*v*_^2^ (half-normal prior distribution [29]).

For model selection, the deviance information criterion (DIC) proposed by [30] was used. This measure provides a trade-off between model fit and complexity. Smaller values of DIC indicate a preferred model. For model selection, the following procedure was used. Using the Poisson log-normal model, covariates were selected in three steps: (1) selection of variables from the univariate models that returned p-value < 0.20; (2) forward selection based on DIC, adding variables individually in descending order of decrease in DIC; and (3) backward elimination from the final model of step (1), removing variables individually in descending order of decrease in DIC.

The Moran I method [31] and CAR model analysis were also performed to detect residual spatial structure.

Calculations required for inference were performed using integrated nested Laplace approximations, a method proposed recently for approximate Bayesian inference in latent Gaussian models. Bayesian p-values were calculated from the predictive posterior probability distribution [32,33]. R software (version 2.15) was used for all data analyses.

The Ethics Committee of the National School of Public Health, Oswaldo Cruz Foundation, approved the project under number 237/2010.

## Results

Overall in the municipality, 2,572 new cases were recorded, a detection rate of 3.70 cases/10,000 population. Women predominated slightly (1.05), together with multibacillary forms (1.02), high detection of cases with grade II physical disability (0.12) and a high number of cases with indeterminate recent form (0.18). The data describes an endemic scenario in the municipality, with neighbourhoods displaying higher percentages of detected cases. When detection rates are considered, however, taking into account the population of each neighbourhood, another scenario emerges.

Of the 40 neighbourhoods in the municipality, detection was higher among women in 25, more paucibacillary cases were detected in 14 and considerable cases with grade II physical disability were detected in four. Finally, cases with indeterminate form (recent infection) accounted for most detections in four neighbourhoods.

**Figure 2:**
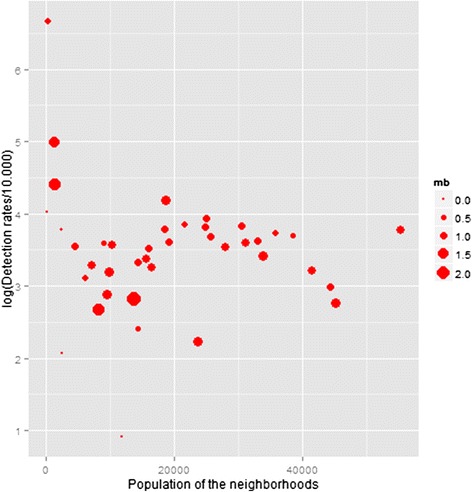
Variability of detection rates by neighborhood.

When analysing only neighbourhoods with the highest detection rates, detection was higher among men and of multibacillary forms. Detection rates show greater variation among neighbourhoods with smaller populations and tend to fluctuate less as population size increases. The ratio between the multibacillary and paucibacillary variables shows that the predominance of multibacillary forms among the cases occurs mainly in neighbourhoods with smaller populations (Figure 2), and most of these neighbourhoods are in areas where health care was decentralised recently, where there may still be hidden prevalence.

From the univariate model analysis (Table 1), the following covariates were selected: proportion of households with running water; number of referral health facilities (with a dermatologist) with a leprosy care programme; ratio of cases with indeterminate clinical form to the sum of the cases with tuberculoid, dimorphous and lepromatous clinical forms; proportion of households where the head has no income; ratio between cases with multibacillary and paucibacillary operational classification; proportion of households with seven or more residents; and number of local case-tracking campaigns performed.

**Table 1:** **Regression models of leprosy detection rates. Duque de Caxias, Brazil, 1998-2006**

**Covariates**	**Univariate poisson log-normal**					**Multivariate poisson log-normal**					**CAR**				
	**BETA**	**CI1**	**CI2**	**P-value**	**DIC**	**BETA**	**CI1**	**CI2**	**P-value**	**DIC**	**BETA**	**CI1**	**CI2**	**P-value**	**DIC**
Proportion of households with general water network	-2.011	-3.514	-0.532	0.008	290.030	-2.334	-3831	-0.851	0.002		-2.333	-3.847	-0.843	0.002	
Number of reference health care units (with a dermatologist) with assistance provided by the Program of Leprosy	0.419	-0.013	0.857	0.057	291.649	0.524	0.092	0.963	0.018		0.524	0.092	0.968	0.018	
Ratio between cases with indeterminate clinical form and the sum of cases with clinical tuberculoid, dimorphous and Virchowian forms	1.901	-0.122	3.980	0.068	290.312	2.599	0.706	4.556	0.008		2.599	0.704	4.588	0.008	
Proportion of households where the head is unemployed	1.155	-0.187	2.491	0.089	292.049										
Ratio between cases with multibacillary and paucibacillary based on operational classification	0.391	-0.087	0.889	0.115	289.815	0.319	-0.141	0.796	0.180		0.319	-0.141	0.803	0.183	
Proportion of households with seven or more residents	0.789	-0.252	1,809	0.132	292.488										
Number of local case-tracking campaigns performed	0.043	-0.020	0.107	0.182	291.166	0.019	-0.042	0.081	0.537		0.019	-0.043	0.082	0.540	
Proportion of households where the head earns up to 1 minimum wage	-0.341	-1.145	0.472	0.406	292.071										
Proportion of households with running water in at least one room	1.223	-3.331	5.823	0.599	291.571										
Proportion of households with general sewage network	0.736	-2.273	3.752	0.630	291.718										
Ratio between cases in female and male genders	-0.082	-0.613	0.465	0.765	292.239										
Log (population density)	-0.028	-0.183	0.131	0.725	292.085										
Ratio between cases with grade II physical disability and the sum of the cases with grades I and 0	0.212	-1.184	1.622	0.766	291.709										
Proportion of households that dispose of waste in vacant lot	8.299	-49391	65924	0.778	291.723										
Proportion of households where the head is illiterate	0.475	-4,907	5.669	0.860	292.114										
Proportion of households without bathroom	-0.015	-12872	12618	0.998	291.943										
Number of basic health care units (with Family Health Programs) assistance provided by the Program of Leprosy	0.014	-0.419	0.451	0.949	291.625										
										285.368					285.544

The final multivariate model included five of these covariates: proportion of households with running water; number of referral health units (with a dermatologist) with a leprosy care programme; ratio between cases with multibacillary and paucibacillary operational classification; and number of local case-tracking campaigns performed. The first three of these covariates returned significant p-values (Table 1). Based on Moran’s I Index, no spatial structure was detected in the residues. Furthermore, the extended CAR model displayed a DIC greater than the DIC returned by the final Poisson log-normal model.

**Figure 3:**
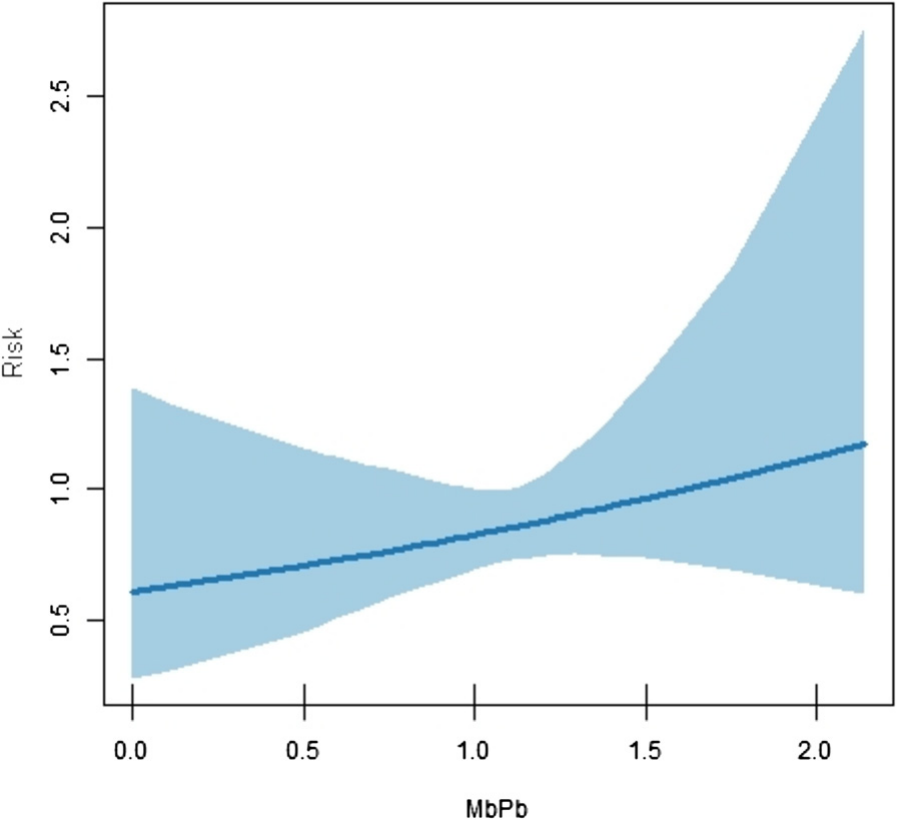
Risk versus ratio between multibacillary and paucibacillary operational classification.

**Figure 4:**
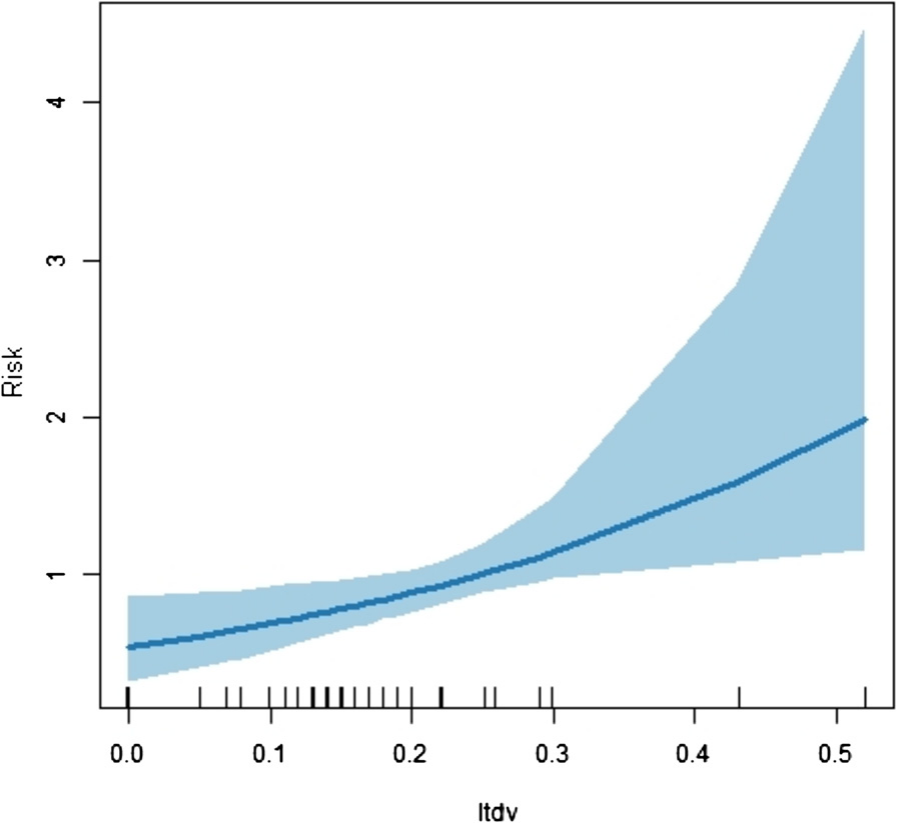
Risk versus ratio between new cases and clinical forms.

As estimated by the final model, the risk of higher case detection declines by 20% when the proportion of households with running water increases by 0.1 (10%). For the covariates ‘ratio of cases with indeterminate clinical form to the sum of cases with tuberculoid, dimorphous and lepromatous clinical forms’ and ‘ratio between cases with multibacillary and paucibacillary operational classification’, graphs were drawn to facilitate interpretation, since these covariates were constructed through ratios and not data percentages (Figures 3 and 4).

The risk of greater detection of new cases can be seen to increase with increases in the covariates ‘ratio between new cases with multibacillary and paucibacillary operational classification’ and ‘ratio of new cases with indeterminate clinical form to other clinical forms’. The more extreme covariate values must be considered with caution, as the confidence interval appears to be stretched (Figures 3 and 4).

## Discussion

The ratio between multibacillary and paucibacillary cases reflects transmission, because it relates to patient ability to eliminate bacillus [2]. It is best interpreted by considering two distinct situations: 1) when the region in question does not actively detect new cases; and 2) when the region invests intensely in actions to combat the disease. In the former situation, little or no action to detect new cases allows patients to remain in the transmission chain for longer periods (greater hidden prevalence). This increases the chance of transmission by higher bacillary load, including by individuals who have stronger immunity to the *M. leprae* bacillus. This reflects in the gap between detection rates and incidence. In such a situation, both paucibacillary and multibacillary cases can be detected in similar proportions, as there are a greater number of recent infection cases occurring in the region. In the latter situation, when strategic action is taken to combat the disease, there is a downward trend in the number of cases, especially the most recent and most symptomatic, and a consequent reduction in the bacillary load and in the chance of transmission (lower hidden prevalence). In such situations, the detection and incidence rates are similar. This leads to a continued presence of mainly oligosymptomatic cases, which are mostly old multibacillary cases with long incubation periods (Figure 1) [34].

The results revealed the latter of the two situations mentioned above, resulting from the trend to early diagnosis, as mentioned in the descriptive analysis of this study, especially increased detection of indeterminate form cases. Recent studies have described improvement in the endemic situation in the municipality resulting from intensified municipal actions over the past decade, with decentralised tracking and treatment of new cases, leading to early diagnosis and reduction of cases with physical disability [35,36]. Therefore, in the final multivariate model, the multibacillary form was substantially associated with higher detection rates. This datum should be interpreted in the context of a decrease in circulating bacilli, secondary to lower hidden prevalence and similar detection and incidence rates. The municipality may be going through a transition in endemic situation, and the higher detection of multibacillary cases may represent older cases. In addition, there was significant economic growth and improvement in sanitary conditions during the study period. The data may reflect a reduction in the endemic similar to that described in Norway [3].

What is interesting is that the early diagnosis covariate (indeterminate clinical form) was also included in the final model, with a significant p-value and strongly explaining the higher detection rates. This reinforces the idea that bacillary load has been reduced and that the trend is towards elimination of the disease in the region [3,21].

Higher detection of new cases was also explained by two covariates that describe strategies in place in the municipality, which are specific to the region. One was the focused campaign, a different proposal carried out in partnership with undergraduate medical students and graduate dermatology students from Rio de Janeiro Federal University. The idea was to concentrate efforts in small areas with higher frequency, instead of performing a single annual campaign covering the whole municipality. In addition, health personnel from local health care facilities involved were also specifically trained. Cavaliere described the contribution of the campaign strategy, showing a higher proportion of detected cases with single lesion and zero-grade physical impairment, as well as a considerable increase in detection of cases among contacts. The campaigns proved to be a tool for early diagnosis and for implementation of contact surveillance [35]. In the adjusted model, however, the campaign’s ability to explain the increase in detection rates was quite low, with a non-significant p-value, possibly as a result of concomitance with actions to decentralise the health service.

The second covariate that describes the municipal actions is the decentralisation of patient care to health facilities with a dermatologist. Higher detection rates showed statistically significant association with neighbourhoods with decentralised facilities. The official recommendation is to decentralise care of leprosy patients to all health facilities, both primary care facilities and facilities with a specialist. The municipality chose to structure a system connecting primary care facilities and facilities with a dermatologist functioning as local region referral centres (decentralised) [37]. Easier access to care for leprosy carriers in the whole municipality probably expanded early diagnosis and, consequently, reduced the number of disabled patients. This facilitation and expansion of case tracking, by democratising diagnosis to a larger number of health care professionals besides specialists, plays a role in the disease elimination process [38]. However, the closer technical support that the referral facilities offered the primary care facilities, for diagnosis and for management of the more complicated cases, may have produced higher quality in local care. In addition, dermatological ambulatory care also allows routine tracking of new cases, especially the oligosymptomatic and/or atypical cases. The importance of facilities with a specialist is mentioned in the National Leprosy Control Programme General Coordination’s management report: “… to integrate primary care with specialised ambulatory and hospital care, offering timely care for all patients…” [23].

## Conclusions

One of the current questions is how effective are the strategies established for the target of eliminating Leprosy as a public health problem [14]. The Health Ministry defines its central strategy as decentralisation of case diagnosis and treatment to all primary health care facilities. However, will this be enough to reach the target? A study in Ceará State [39] showed that decentralisation plays an important role, but does not itself improve health system performance in providing care. In our study, the data on the municipality point to a possible positive association between the interventions performed in the municipality and the changes in the epidemiological situation of leprosy.

Strategies to combat the disease by targeting asymptomatic (possibly bacilliferous) individuals must be expanded beyond using the BCG vaccine to boost the immune response, as is currently being done. If primary care routines were to include specific immunological tests to identify antibodies against *M. Leprae*, that could permit better knowledge and control of the silent individual perpetuators in the disease transmission chain, whose role has been to retard elimination of the disease in many regions [40].

If country data shows progress towards elimination of the disease, attention must now increasingly be focused on sub-country and sub-municipal areas. The data in this study show endemic heterogeneity within the municipality. Analysis and precise identification of these more critical areas must be encouraged. In addition, the SINAN database system must be improved, with more emphasis on specifying neighbourhoods, districts and other municipal subdivisions, as well as census tracts. This could allow interventions that are more specific to the area context.

One limitation of this study is that variables “expected” to be significant in the final model, along with the presence of the spatial terms, are not contemplated due to the high degree of data aggregation (neighbourhoods) and small sample size.

Although the results indicate progress towards eliminating the disease from the Duque de Caxias municipality, high detection rates persist in municipal sub-regions. In that light, the following question can be posed: to what point, or rather, in how wide a geographical area, is it possible to talk about real elimination of the disease, given heterogeneity within a municipality of only 442sq km? Leprosy must thus be controlled with the same intensity once the municipal prevalence rate target of less than 1 case for every 10,000 inhabitants has been reached, with strategic actions directed especially at areas with the highest rates within the municipality itself.
